# Childhood cancer and magnetic fields from high-voltage power lines in England and Wales: a case–control study

**DOI:** 10.1038/sj.bjc.6605795

**Published:** 2010-09-28

**Authors:** M E Kroll, J Swanson, T J Vincent, G J Draper

**Affiliations:** 1 University of Oxford, Childhood Cancer Research Group, Richards Building, Old Road Campus, Headington, Oxford OX3 7LG, UK; 2 National Grid, 1-3 Strand, London WC2N 5EH, UK

**Keywords:** childhood leukaemia, childhood cancer, magnetic fields, power lines

## Abstract

**Background:**

Epidemiological evidence suggests that chronic low-intensity extremely-low-frequency magnetic-field exposure is associated with increased risk of childhood leukaemia; it is not certain the association is causal.

**Methods:**

We report a national case–control study relating childhood cancer risk to the average magnetic field from high-voltage overhead power lines at the child's home address at birth during the year of birth, estimated using National Grid records. From the National Registry of Childhood Tumours, we obtained records of 28 968 children born in England and Wales during 1962–1995 and diagnosed in Britain under age 15. We selected controls from birth registers, matching individually by sex, period of birth, and birth registration district. No participation by cases or controls was required.

**Results:**

The estimated relative risk for each 0.2 *μ*T increase in magnetic field was 1.14 (95% confidence interval 0.57 to 2.32) for leukaemia, 0.80 (0.43–1.51) for CNS/brain tumours, and 1.34 (0.84–2.15) for other cancers.

**Conclusion:**

Although not statistically significant, the estimate for childhood leukaemia resembles results of comparable studies. Assuming causality, the estimated attributable risk is below one case per year. Magnetic-field exposure during the year of birth is unlikely to be the whole cause of the association with distance from overhead power lines that we previously reported.

The possible health effects of exposure to electric and magnetic fields have been extensively discussed. Extremely-low-frequency (ELF) magnetic fields are classified as ‘possibly carcinogenic to humans (group 2B)’ on the basis of ‘limited evidence in humans for the carcinogenicity of ELF magnetic fields in relation to childhood leukaemia’ (IARC Working Group on the Evaluation of Carcinogenic Risks to Humans, 2002, p 338). The World Health Organisation concludes that ‘consistent epidemiological evidence suggests that chronic low-intensity ELF magnetic-field exposure is associated with an increased risk of childhood leukaemia’, although ‘the evidence for a causal relationship is limited’ by potential flaws in the epidemiological studies (particularly control selection bias and exposure misclassification), and a lack of laboratory or mechanistic evidence (World Health Organization, 2007, pp 354–355).

Most individual epidemiological studies on childhood leukaemia have produced relative risk (RR) estimates above 1 for children exposed to fields above 0.20 or 0.25 *μ*T, compared with all other children or with those exposed to low levels (UK National Radiological Protection Board (NRPB), 2001, p 135). A pooled analysis of individual records, from nine studies that met specified quality criteria, found a two-fold increase in risk of childhood leukaemia with residential magnetic-field exposure over 0.4 *μ*T compared with exposures below 0.1 *μ*T (Ahlbom *et al*, 2000).

In 2005, we published results from a case–control study of children born in England and Wales between 1962 and 1995 (Draper *et al*, 2005a). We found a statistically significant positive association between childhood leukaemia and distance from home address at birth to high-voltage overhead power lines. The association extended too far from the power lines to be readily explained by magnetic fields.

In the present study, we obtain, as far as possible, an estimate of the magnetic field derived from high-voltage power lines for the address of each child included in the distance study, and relate this directly to the risk of childhood cancer.

## Materials and methods

### Cases and controls

The study population consisted of children born in England and Wales during 1962–1995. Cases were defined as children who were born in England and Wales during 1962–1995, diagnosed with cancer under the age of 15 in England, Wales, or Scotland during 1962–1995, and recorded in the National Registry of Childhood Tumours, a high-quality population-based specialist childhood cancer registry (Stiller, 2007).

‘Cancer’ was defined as malignancy of any site, together with intracranial and intraspinal tumours of benign and uncertain behaviour. Cases were classified according to the second edition of the International Classification of Childhood Cancer (Kramárová and Stiller, 1996) and grouped into the following three categories: leukaemia, intracranial and intraspinal (‘CNS/brain’) tumours, and other cancers including lymphoma.

Excluding children with known NHS numbers showing that they were born outside England and Wales, there were 32 968 potentially eligible cases. From the Office for National Statistics, we obtained birth information for 31 273 cases; records were not available for 912 cases (e.g., because born abroad, or adopted) and not traced for 783 cases (e.g., because born in Scotland). From birth registers, we selected one control child for each case, matched for sex, date of birth (within 6 months), and birth registration district.

### Assessment of exposure

To be consistent with the pooled analysis by Ahlbom *et al* (2000), we defined the reference category as exposure <0.1 *μ*T.

We attempted to obtain an estimate of the 1-year average magnetic field from high-voltage overhead power lines experienced by each child at the address at birth during the year of birth. We included all the high-voltage overhead power lines of the National Grid in England and Wales. This comprises all overhead power lines that carry the highest voltages used in England and Wales (275 and 400 kV), and the few 132 kV lines (<1% of the total at this voltage, by length) that form part of the National Grid rather than regional distribution networks. We had no data for any other power lines. The method of estimation is summarised below. Full details are given in a separate paper (Swanson, 2008).

We had previously (for the distance study (Draper *et al*, 2005a)) calculated the distance from each child's home at birth to any adjacent lines constructed before or during the year of birth. Briefly, if the postcode centroid for the home address was more than 1 km from the line, the distance was defined as >600 m; otherwise the distance was calculated explicitly, using coordinates for the home based on Ordnance Survey ADDRESS-POINT data (2001 Crown copyright), and coordinates supplied by National Grid for the 21 800 pylons that define the routes taken by the lines, supplemented by detailed mapping, and some site visits for homes within 50 m of the line. Postcodes could not be determined for 2.5% of cases (786 out of 31 273) and 3.6% of controls (1118 out of 31 273); address-pointing within 1 km failed for 8% of cases (206 out of 2654), and 7% of controls (180 out of 2587). After exclusions for missing distance data and incomplete sets, there were 29 081 complete matched case–control pairs. The mean distance between the case and control addresses in each matched pair was 11 km.

We used the National Grid computer programme EM2D to calculate estimates of the magnetic fields produced by power lines in nearby homes. The physical model underlying the programme has been validated by comparing the actual magnetic fields produced by real power lines, under known currents, with the fields predicted by the model for the same lines and currents (Swanson, 1995). Under operating conditions relevant to this study, the field predicted by the model is considerably below 0.1 *μ*T at any address that is beyond 200 m from a line that has transposed phasing, or beyond 400 m from a power line of any type. Therefore, all study addresses with these characteristics (57 704 of 58 162, i.e., 99% Table 1) were assigned to the reference category without explicit field calculation.

The distance from the power line is an important factor in the calculation. The programme also requires detailed input data on characteristics of the power line such as phasing and current. Specifications that had not changed over time were readily available from National Grid records. Other data were difficult to obtain, particularly for birth years in the earlier part of the study (Swanson, 2008). The average currents carried by each line in the relevant years had to be estimated from records of predictions for peak current made (up to 5 years in advance) by National Grid for planning purposes. Data were missing or very unreliable for 25% of the addresses that were close enough to a power line to be candidates for field calculation (116 out of 458; Table 1). These addresses, and their matched partners, were excluded from the magnetic-field analysis.

### Statistical analysis

After exclusions for missing magnetic-field data and incomplete sets, as described above, there were 28 968 complete matched case–control pairs: 9653 for leukaemia, 6584 for CNS/brain tumours, and 12 731 for other cancers.

We analysed the data by conditional logistic regression, using standard methods for an individually matched case–control study, and taking the odds ratio as an estimate of RR (appropriate because childhood cancer is very rare). Following the specifications chosen by Ahlbom *et al*, we estimated the RR separately for each of the exposure categories 0.1 to <0.2 *μ*T, 0.2 to <0.4 *μ*T, and ⩾0.4 *μ*T, taking exposures below 0.1 *μ*T as the reference category. Following Ahlbom *et al*, we also fitted the risk as a continuous linear function (on the log scale) of the estimated field at each individual address (setting exposures below 0.1 *μ*T to 0), to obtain an estimate of the proportionate increase in risk for an increase in exposure of 0.2 *μ*T.

To investigate whether characteristics of power-line routes might affect the results, we repeated the analysis with adjustment in turn for the socioeconomic status (quintile categories within England and Wales of the Carstairs deprivation score (Morris and Carstairs, 1991)), population density, and urban–rural status of the census ward of each child's address, using 1981 census data that had been obtained from the University of Manchester for an earlier study.

## Results

The relationship between distance and estimated magnetic field is summarised in Table 1 for the birth addresses of the 58 162 children included in our original distance study. In total, 1712 addresses were within 600 m of a high-voltage power line. The annual-average magnetic field could reliably be assumed to be <0.1 *μ*T (the upper limit of the reference category) at 1254 of these addresses (73% of 1712). We attempted to estimate the field for each of the remaining 458 addresses. The field could not be estimated for 116 addresses (25% of 458). There was no evidence for a difference between cases and controls in the proportion of attempted estimates that failed: this proportion was almost identical (approximately 32%) for cases and controls in the leukaemia group, and did not differ significantly between cases and controls in either of the other two diagnostic groups (*P*=0.095 for CNS/brain tumours and *P*=0.87 for ‘other cancer’) (Table 2). The field was estimated to be <0.1 *μ*T for 299 addresses (65% of 458), and >0.1 *μ*T for 43 addresses (9% of 458); the latter were all within 156 m of a power line.

Table 3 shows the distribution of subjects by case–control status, diagnostic group, and magnetic-field category, after excluding addresses for which no field estimate could be obtained, and the matched partners of those addresses. There were 21 cases and 22 controls with estimated magnetic fields >0.1 *μ*T.

At the 5% significance level, none of the estimated RRs differed from unity (Table 4).

For childhood leukaemia, the estimated risk relative to the reference category was 2.00 in both the highest and the lowest exposure categories, and there were no cases in the intermediate category. The estimated RR associated with each 0.2 *μ*T increase in magnetic field was 1.14 (95% confidence interval 0.57 to 2.32); this model did not fit significantly better than the null model (*P* for trend=0.70).

There was no suggestion of excess risk for CNS/brain tumours. We assume that the high RR estimate for ‘other cancers’ in the highest exposure category is a chance finding, as we had no previous evidence for such an association in this specific group. The five cases in the highest exposure category were all of different diagnostic types: retinoblastoma, nasopharyngeal carcinoma, malignant gonadal germ-cell tumour, bone sarcoma, and neuroblastoma. The estimated RR for each 0.2 *μ*T increase in magnetic field was 0.80 (0.43–1.51) for CNS/brain tumours (*P* for trend=0.20), and 1.34 (0.84–2.15) for other cancers (*P* for trend =0.13).

Adjustment for socioeconomic status, population density, or urban–rural status made very little difference to the results (not shown). The estimated RR for each 0.2 *μ*T increase in field became 1.13 (0.56 to 2.29) for leukaemias when adjusted for socioeconomic status; the effects of other adjustments were even smaller.

## Discussion

We found no statistically significant associations between childhood-cancer risks and estimated magnetic fields from high-voltage power lines near the child's home address at birth. The risk estimate for leukaemia slightly strengthens the existing evidence for a positive association in this diagnostic group.

### Strengths of the study

This is a large study based on a high-quality population-based cancer registry, with exposures estimated from records held by the national electricity transmission utility. Exposure categories were defined in advance, exposure allocation was blind to case–control status, and there was no active participation by cases or controls. Hence, various potential sources of bias were avoided.

The study obtains estimates of magnetic field from power lines. In a home that is close to a high-voltage power line, the power line is the most important source of magnetic field. Where the power-line field is at least double the size of the field from other sources, the combined field in the home is on average no more than approximately 10% above the power-line field alone (Swanson, 2008). In the United Kingdom, the mean magnetic field in homes is approximately 0.05 *μ*T (mainly from low-voltage distribution wiring) (Swanson and Kaune, 1999; UK Childhood Cancer Study Investigators, 2000b). So, on average, above 0.1 *μ*T (the boundary of the reference category in this study) the power-line field should be a good approximation to the overall magnetic field in the home.

### Limitations of the study

We made no attempt to estimate magnetic fields from sources other than high-voltage power lines, such as substations, underground cables, distribution or house wiring, or domestic appliances. This is a potential source of exposure misclassification for children whose addresses were not close to power lines. However, in another case–control study set in Britain, 2.3% of control children were exposed to magnetic fields (from any source) above 0.2 *μ*T (UK Childhood Cancer Study Investigators, 1999); this level of field was due to a high-voltage overhead power line in 9 (9%) of 102 surveyed homes (Maslanyj *et al*, 2007). This suggests that not more than approximately 2% of children in our ‘unexposed’ group would have experienced fields above 0.2 *μ*T at the birth address from other sources, so that any resulting bias in the estimate of risk should be very small.

The main limitation of the study is the incompleteness of the existing records of operating characteristics of power lines in the past. The most important factor, distance, was accurately known, but there was considerable uncertainty in some of the other data. The sensitivity of the magnetic-field calculations to inaccuracies in the input data is assessed elsewhere (Swanson, 2008). Inaccuracies in the magnetic-field estimate would be unlikely to lead to differential exposure misclassification of cases and controls, but would tend to move the estimated trend in RR towards the null (Rothman and Greenland 1998, p 130).

Relatively few children had magnetic-field estimates above the reference level. Exposures were generally lower during the early part of the study than in recent years, because both the proportion of addresses near the lines and the currents carried by the lines were lower; moreover, fields could not be estimated for some addresses near power lines because of missing data. Hence, the confidence limits on the risk estimates are quite wide, in spite of the very large total number of children (28 968 case–control pairs).

### Comparison with previous studies of magnetic-field exposure as a risk factor for childhood leukaemia

Unlike most previous studies of the same topic, our study assesses exposure during the year of birth, and not during later childhood (up to the time of diagnosis). There is no convincing theoretical reason to prefer either approach to the other, as there is no known mechanism for an effect of magnetic fields, and initiating or promoting events might occur in gestation, during childhood, or both. Some childhood cancers are undoubtedly prenatal in origin. For approximately half the cases (48% of leukaemias, 44% of CNS/brain tumours, and 49% of other cancers) the address at diagnosis was the same as the address at birth; we have no information on the addresses of controls at the time of diagnosis.

The UK Childhood Cancer Study (UKCCS) calculated magnetic fields from measured currents in overhead power lines with voltages ranging from 11 to 400 kV, for the home addresses of 6770 children in Britain during 1991–1996 (3380 cases of childhood cancer and 3390 controls) (UK Childhood Cancer Study Investigators, 2000a). The geographical areas and time periods overlap with those for our study, and the same computer programme was used for the field calculations, but the overall proportion of addresses with fields above 0.1 *μ*T is higher in the UKCCS (0.52%) than in our study (0.07%). Various differences between the studies help to explain this apparent discrepancy. In particular, line currents were generally higher during the periods covered by the UKCCS than in the early years of our study, and the UKCCS includes additional (lower-voltage) power lines. Confining the comparison to the 275 and 400 kV lines, and to the part of our study that overlaps in time with the UKCCS, the proportion of addresses with calculated magnetic fields above 0.1 *μ*T is 0.21% in the UKCCS (Prof E Roman, personal communication; consistent with (Maslanyj *et al*, 2007)), and is estimated to be 0.23% in our study during 1991–1995. We conclude that the proportions of addresses with fields above 0.1 *μ*T in the two studies are not incompatible.

Most of the existing evidence regarding a possible association between magnetic fields and childhood leukaemia is summarised in the pooled analysis by Ahlbom *et al* (2000). Two different types of study were included: those that used calculated fields from power lines to represent the overall field in the home (based on the latest address in the power-line corridor before diagnosis, or the address during the last year before diagnosis), and those that used measurements of the magnetic field in the home from all sources. When pooled separately, the two types of study produced similar results, although they were potentially subject to different types of inaccuracy, confounding and bias; this makes it less likely that artefact is the whole explanation. Overall, Ahlbom *et al.* estimated a RR of 2.00 (1.27 to 3.13) for the over 0.4 *μ*T category compared with the under 0.1 *μ*T category, and a RR of 1.15 (95% confidence interval 1.04 to 1.27) for each 0.2 *μ*T increase in magnetic field. The results of subsequent studies (e.g., Schüz *et al*, 2001; Kabuto *et al*, 2006) have been consistent with these estimates. From the calculated-fields studies alone, Ahlbom *et al.* estimated a RR for childhood leukaemia of 1.11 (95% confidence interval 0.94 to 1.30) for each 0.2 *μ*T increase in magnetic field, based on the address at or before diagnosis. This is similar to the corresponding risk estimate from our study, based on address at the time of birth, which was 1.14 (0.57 to 2.32).

### Comparison with risk estimates based on distance from power lines

Our case–control study of distance from high-voltage power lines (Draper *et al*, 2005a) estimated the RR of childhood leukaemia as 1.69 (95% confidence interval 1.13 to 2.53) and 1.23 (1.02 to 1.49), respectively, for children whose birth addresses were within 200 m and between 200 and 600 m from high-voltage power lines, compared with those beyond 600 m. We noted that calculated fields beyond 200 m from power lines are usually <0.1 *μ*T, and often <0.01 *μ*T, and concluded that we had no satisfactory explanation of the results in terms of magnetic fields.

**Figure 1:**
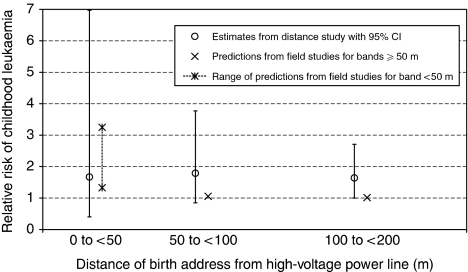
Estimated RR of childhood leukaemia by distance of birth address from high-voltage overhead power line (see text). Reference levels: distance ⩾600 m, magnetic field <0.1 *μ*T.

To investigate the plausibility of a magnetic-field explanation within 200 m of the power line, we compared RR predictions based on field estimates from this study with RR estimates taken from the distance study, for the distance bands 50 to <100 m and 100 to <200 m. We applied Ahlbom's continuous RR estimate (1.15 per 0.2 *μ*T) to the field estimate for each control address within the band (using controls from all three diagnostic groups), and then took the mean of these predictions as the ‘field risk’ within that band. (We chose Ahlbom's rather than our own similar RR estimate because it is more precise.) We obtained an approximate 95% confidence interval for the ‘field risk’ in each of the two bands by estimating its variance as a function of the variance of the field estimates within the band and of the variance of Ahlbom's RR. The resulting ‘field risks’ are 1.05 (95% confidence interval 1.01 to 1.10) and 1.01 (1.00 to 1.03), respectively. The corresponding RR estimates from the distance study were 1.79 (0.85 to 3.77) and 1.64 (0.996 to 2.71), respectively. Thus, within each of the distance bands 50 to <100 m and 100 to <200 m, the mean RR prediction from estimated magnetic-field exposure is much smaller than the actual RR estimate found by the distance study. This argues against a magnetic-field explanation of the distance association in these bands, but the predictions are not strictly incompatible with the distance-study estimates, given the wide confidence intervals of these estimates (Figure 1).

We did not feel confident in applying the Ahlbom continuous risk estimator to one very high field value (4.71 *μ*T) occurring in the closest distance band (0 to <50 m). So, for this band, we have calculated two alternative RR predictions, the first using the Ahlbom model, as for the other two distance bands, and the second assuming that this model applies only up to fields of 1 *μ*T, and that the risk does not increase any further at higher levels. This sensitivity analysis gives a RR prediction in the range 1.33 to 3.25. This is clearly consistent with the estimate for this band from the distance study, which was 1.67 (95% confidence interval 0.40 to 6.97) (Figure 1).

We conclude that exposure to magnetic fields from high-voltage power lines at the birth address during the year of birth is extremely unlikely to be the whole cause of the apparent increase in childhood leukaemia risk observed in our earlier case–control study, which assessed the distance of the birth address from these lines. We were unable to investigate any possible effect of duration of exposure between birth and diagnosis, since we had no residential history for the controls after birth. Other potential explanations (instead of, or in addition to, magnetic fields) include chance, confounding, or some unidentified causal factor related to power lines (Draper *et al*, 2005b; Swanson *et al*, 2006).

### Attributable risk

The proportion of cases of disease D attributable to a specified causal exposure E in a population (the ‘attributable risk’) can be expressed as AR=((RR–1)/RR) × Pr(E∣D), where RR is the relative risk and Pr(E∣D) is the probability that a case has this exposure.

If we assume that exposure to magnetic fields is a cause of childhood leukaemia, we can estimate the attributable risk from the field estimates made for this study, with the categorical RR estimates shown in Table 4. (For one of the three magnetic-field bands, 0.2 to <0.4 *μ*T, the RR could not be estimated, as no case had a field estimate in this band). For each field band, we used the proportion of cases lying within the band to estimate Pr(E∣D), making allowance for missing field estimates. The total AR is 0.0006. Thus, we estimate that, for the period covered by this study, exposure during the year of birth to magnetic fields from high-voltage power lines of the type included here could have caused approximately 6 in 10 000 childhood leukaemia cases in England and Wales, or one case approximately every 4 years.

In more recent years, both power-line loads and the numbers of dwellings near to lines have increased. For example, as reported above, the proportion of birth addresses exposed to fields above 0.1 *μ*T during 1991–1995 was roughly three times the average for the whole study. If we assume that this ratio applies in each field band, the attributable risk will increase by approximately the same factor. The revised estimate is still only approximately 1 in 500 cases, that is, approximately 1 case per year.

## Conclusions

This study slightly strengthens the existing evidence for an association between magnetic fields and childhood leukaemia. However, magnetic fields during the year of birth are extremely unlikely to be the whole cause of the apparent increase in childhood leukaemia risk observed in our previous case–control study of the distance of the birth address from these lines. We emphasise that the fact that very few homes in Britain are exposed to high magnetic fields from power lines means that, for the population as a whole, the public-health risk from such exposure would be very small.

## Figures and Tables

**Table 1:** Number of addresses by distance from power line and type of magnetic-field estimate: all cases and controls included in the distance study

	**Magnetic-field estimate (*μ*T)**						
		**Calculated**					
**Distance (m)**	**Assumed <0.1**	**<0.1**	**0.1 to <0.2**	**0.2 to <0.4**	**⩾0.4**	**None**	**Total**
⩾600	56 450	0	0	0	0	0	56 450
500–599	390	0	0	0	0	2	392
400–499	398	0	0	0	0	1	399
300–399	254	45	0	0	0	24	323
200–299	211	53	0	0	0	27	291
100–199	1	162	3	1	0	38	205
50–99	0	37	15	2	0	17	71
<50	0	2	5	4	13	7	31
							
Total	57 704	299	23	7	13	116	58 162

**Table 2:** Numbers of cases and controls by status of magnetic-field estimate, with result of Fisher's exact test for association (*P*): distance-study addresses (within 400 m of power line) for which field estimation was attempted

	**Magnetic-field estimate**			
**Diagnostic group**	**Succeeded**	**Failed**	**Total**	***P***
*Leukaemias*				1.00
Cases	59	28	87	
Controls	37	18	55	
				
*CNS/brain* *tumours*				0.095
Cases	47	6	53	
Controls	48	15	63	
				
*Other cancers*				0.87
Cases	73	23	96	
Controls	78	23	101	

**Table 3:** Numbers of cases and controls by magnetic-field category and diagnostic group: all cases and controls included in the magnetic-field study

	**Magnetic field (*μ*T)**				
**Diagnostic group**	**<0.1**	**0.1 to <0.2**	**0.2 to <0.4**	**⩾0.4**	**Total**
*Leukaemias*					
Cases	9645	6	0	2	9653
Controls	9647	3	2	1	9653
					
*CNS/brain tumours*					
Cases	6580	2	1	1	6584
Controls	6577	4	0	3	6584
					
*Other cancers*					
Cases	12 722	2	2	5	12 731
Controls	12 722	6	2	1	12 731
					
Total	57 893	23	7	13	57 936

**Table 4:** Relative risk estimates (95% confidence interval) by magnetic-field category, and with magnetic-field exposure as a continuous variable

	**Categorical analysis. Reference level: < 0.1 *μ*T**			
	**Magnetic field (*μ*T)**			**Continuous analysis**
**Diagnostic group**	**0.1 to <0.2**	**0.2 to <0.4**	**⩾0.4**	**RR per 0.2 *μ*T**
Leukaemias	2.00 (0.50–7.99)	0 case/ 2 controls	2.00 (0.18–22.04)	1.14 (0.57–2.32)
CNS/brain tumours	0.50 (0.09–2.73)	1 case/ 0 control	0.33 (0.03–3.20)	0.80 (0.43–1.51)
Other cancers	0.33 (0.07–1.65)	1.00 (0.14–7.10)	5.00 (0.58–42.80)	1.34 (0.84–2.15)

Abbreviation: RR=relative risk.
